# Miscellany

**Published:** 1905-09

**Authors:** 


### Miscellany.

Caution: Death from Blood-Poisoning, following Local Anæsthesia for Tooth Extraction.—A case is recorded in the *British Journal of Dental Science* for July 15, 1905, page 664, that deserves more than a passing notice. A woman called upon "an artificial tooth-maker" (this is a new vocation, having its origin in the peculiar provision of the British Dental Act, which forbids those not legally qualified assuming the name of "dentist" without forbidding them to practise the art. To evade this act, the unqualified call themselves "tooth-makers" and "tooth-extractors") for the purpose of having several teeth extracted. On the first visit six were removed, and three on a subsequent visit a week later. On both occasions "White's" anæsthetic was injected with a hypodermic syringe. About a week after the second visit, suffering pain in her head and being unable to open her mouth, she called upon a physician for relief. She was then suffering from lockjaw, which continued to the end. Five or six days later a large abscess developed upon the jaw. This was promptly opened and freely drained; notwithstanding this, she developed an acute attack of blood-poisoning, from which she died. At the inquest the operator testified that at the second visit there was a little matter (*i.e.*, pus) around the seat of the previous extractions; that while the wound was not entirely healed, it did not seem to be in a serious condition. He testified that he kept his needles in alcohol to keep them from rusting, and boiled them fifteen minutes before using them again. The testimony seemed to show that the untoward result was due to pus present on the gums near the site of the first extractions being injected into the gums with the anæsthetic used preparatory to the second operation.

Notwithstanding that it was shown that the operator had commenced practice after only six months' instruction, the woman's death was not charged to his incompetency. This was doubtless just, from the fact that the importance of sterilizing the field of operation immediately before making a hypodermic injection is not generally appreciated, nor yet is it generally practised by dentists.

The jury returned a verdict of "Death from blood-poisoning," adding that there was nothing to show how the poison had been absorbed.

It is remarkable that so few cases like this have been recorded, since hypodermic injections for tooth extraction has become so general, and the precautions adopted to avoid this danger are usually limited to sterilizing the needle. Would not a like result be liable to occur were one of a series of teeth or tooth-roots subjected to this treatment preparatory to extraction abscessed?—W. H. T.

---

For cleansing bridges and regulation apparatuses a saturated solution of caustic soda will prove very efficient. It will render them aseptic. (*Le Laboratoire.*)—C. M. L.

